# Receptor-interacting protein kinase 2 is associated with tumor immune infiltration, immunotherapy-related biomarkers, and affects gastric cancer cells growth *in vivo*

**DOI:** 10.7150/jca.90008

**Published:** 2024-01-01

**Authors:** Qian Yang, Kunqiao Hong, Yu Li, Pengshuang Shi, Fang Yan, Peng Zhang

**Affiliations:** 1 Department of Gastroenterology, Guizhou Provincial People's Hospital, Medical College of Guizhou University, Guiyang City, Guizhou Province, PR China.; 2 Department of Gastroenterology, Renmin Hospital of Wuhan University, Wuhan City, Hubei Province, PR China.; 3 Department of Urology, Guizhou Provincial People's Hospital, Guiyang City, Guizhou Province, PR China.

**Keywords:** RIPKs, immune infiltration, immune checkpoint, tumor stemness, RNA methylation

## Abstract

**Background:** The objective of this study was to analyze the research trend of four RIPK genes (RIPK1, RIPK2, RIPK3, and RIPK4), their expression variations in tumors, and the correlation between RIPK2 expression and immune-related biomarkers in gastric cancer (GC).

**Methods:** The PubMed database was utilized to investigate the research trend surrounding four RIPKs genes in tumors. The ULCAN database was employed to analyze the differential expression of these four RIPKs genes. TCGA data were utilized to examine the association between RIPK2 expression and various factors including tumor immune infiltration and immune-related biomarkers. Lastly, the impact of targeting RIPK2 on the growth of GC cells was confirmed through tumor formation assay, immunohistochemistry, and Tunnel assays.

**Results:** In the field of tumor biology, there has been a sustained increase in research focused on the four RIPKs genes over the past decade. Four RIPKs genes are differentially expressed in a majority of tumors. Furthermore, this investigation has unveiled a connection between the expression of RIPK2 and the infiltration of four immune cells, as well as the presence of RNA methylation modifying enzymes, specifically m1A, m6A, and m5C, in GC. Additionally, RIPK2 expression was associated with the genes related to immune checkpoint regulation, as well as genes associated with immunoinhibitors and immunostimulators. It was also revealed that RIPK2 expression was correlated to immunotherapy response biomarkers, namely MSI and TMB, and tumor stemness. Ultimately, it was demonstrated that targeting the RIPK2 effectively regulated GC cells growth through the suppression of PCNA expression and the induction of apoptosis.

**Conclusion:** The expression of RIPK2 is correlated with immune cell infiltration, RNA methyltransferase activity, tumor stemness, checkpoint-related genes, and immunotherapy-related biomarkers. Suppression of RIPK2 impedes the growth of GC cells *in vivo*. Consequently, RIPK2 holds promise as a viable immunotherapy target for various types of cancer.

## Introduction

Cancer is a prominent contributor to mortality rates and a substantial impediment to global life expectancy improvement 1. According to the Global Cancer Statistics 2020 report, prostate cancer, lung cancer, colorectal cancer, and liver cancer are the most prevalent malignancies in men, while breast cancer and cervical cancer are the most frequently diagnosed cancers in women 2. In recent times, immunotherapy has emerged as a prominent area of research due to advancements in the understanding of cancer cell immune recognition and immune regulatory molecules 3. Immune checkpoint regulators, known as critical modulators of the immune system, play a pivotal role in eliciting co-stimulation or co-inhibition of T-cell responses. By blocking these checkpoints, the immune system can be empowered to effectively identify and eliminate cancer cells, leading to robust anti-tumor responses 4. Consequently, investigating genes that target immune cells, immune modulators, and immune checkpoint regulators holds promise for augmenting the efficacy of immunotherapy.

Receptor-interacting protein kinase (RIPK) family genes have been found to be of significant importance to the innate immune system, as they play a role in modulating the progression of immune-related diseases and tumors 5-11. Dysregulation of the innate immune system can lead to immunodeficiency or autoinflammatory diseases. Previous research has demonstrated the vital role of RIPK1 in maintaining immune homeostasis 12, and there have been reports of patients with RIPK1-associated immunodeficiency or autoinflammatory diseases 13-16. Furthermore, RIPK1 has been shown to regulate the development of liver cancer 17, pancreatic carcinoma 18, cervical cancer 19 and breast cancer (BRCA) 20. RIPK2 plays a crucial role in intracellular signal transduction pathways, encompassing inflammation, autophagy, programmed cell death, and cancer. Consequently, it is essential for the early control of various pathogenic organisms and the early detection of tumors 21-26. Additionally, a study conducted by Zhang et al. 24 demonstrated that RIPK2 regulates the progression of tumor-infiltrating myeloid-derived suppressor cells, which highlighting the significant role of RIPK2 in tumor immune. RIPK3 has been found to modulate the lipid metabolic reprogramming of tumor-associated macrophages, thereby regulating the immunometabolism 27 and facilitating immune evasion 28 in hepatocellular carcinoma (HCC). The upregulation of RIPK3 has been shown to increase the production of immunostimulatory cytokines in the tumor microenvironment, leading to a robust cytotoxic anti-tumor immune response 9. Additionally, RIPK4 has been identified as an immune regulating-associated biomarker in ovarian cancer 11, 29, where it plays a role in regulating the growth of squamous cell carcinoma 30 and cell-cell adhesion in epidermal development and homeostasis 31. All of the aforementioned studies have demonstrated the significant role of RIPKs genes in the regulation of immune-related diseases, such as tumors. Therefore, we will conduct an analysis focused on immune-related factors.

In addition to fibroblasts, endothelial cells, and stromal cells, the tumor microenvironment (TME) consists of both innate and adaptive immune cells, with infiltrating immune cells representing a substantial proportion 32. Notably, tumor-associated macrophages contribute to immune evasion, tumor angiogenesis, and metastasis. Furthermore, the TME contains macrophages, CD4+ T cells, B cells, CD8+ T cells, and neutrophils, which are involved in the development of cancer 33. In this study, we conducted a preliminary analysis of the role of four RIPK family genes in tumors, with a specific focus on examining the correlation between RIPK2 and tumor immune cell infiltration, immune-related biomarkers, tumor heterogeneity, and tumor stemness in GC. Finally, *in vivo* experiments provided further validation of the inhibitory effect of RIPK2 on GC tumor growth.

## Methods

### Researches on RIPK family genes in cancers

**Figure 1:**
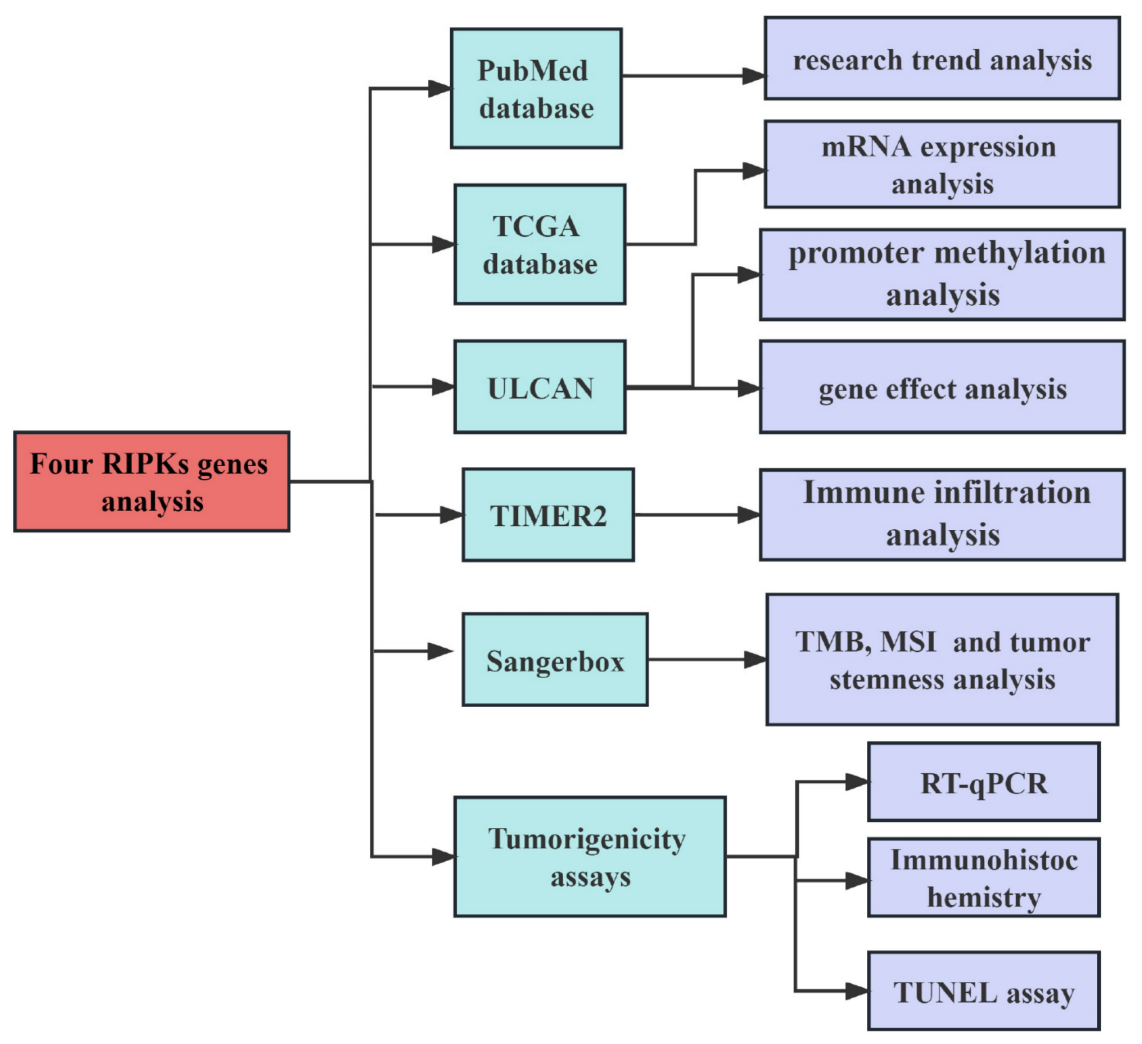
The flowchart of the research process.

We searched articles from the PubMed database (https://pubmed.ncbi.nlm.nih.gov/) on May 8, 2023. The search strategy employed in this study as: (TS= ("RIPKs") AND (TS= ("cancer"). It should be noted that the information obtained from public databases was not comprehensive, as we focused solely on the research information pertaining to four RIPK family genes (RIPK1, RIPK2, RIPK3, and RIPK4). We included the records without time restrictions and article type, but the publishing language was limited to English. Two independent researchers reviewed the titles and abstracts to include the data. Subsequently, we analyzed the mRNA expression levels of RIPK family genes in human tumors and adjacent tissues using data from TCGA 34 (https://portal.gdc.cancer.gov/) data using the Sangerbox tool (http://sangerbox.com/Tool). RIPKs mRNA expression level was used log_2_ (Transcripts per million ^+^ 1) in log-scale. The flowchart of the research process in this study is shown in** Figure 1**.

### mRNA expression and promoter methylation level of RIPKs in gastric cancer

The mRNA expression levels of RIPK1, RIPK2, RIPK3, and RIPK4 in gastric cancer (GC) tissues were investigated using ULCAN 35 a web resource that facilitates the analysis of cancer OMICS data. Additionally, the gene effect scores for RIPKs in gastric cancer cell lines were examined. These scores were obtained from CRISPR knockout screens conducted by the Broad's Achilles and Sanger's SCORE projects 36. The integration of the datasets from Broad and Sanger was carried out following the methodology outlined in prior research 37. Negative scores indicate the inhibition of cell growth resulting from the knockout of specific genes. These scores are standardized, with nonessential genes having a median score of 0, while commonly identified essential genes have a median score of -1.

### Correlations between RIPK2 expression and immune cells infiltration

TIMER2 (http://timer.cistrome.org/) 38 is a comprehensive resource for systematic analysis of immune infiltration across diverse cancer types. The R software "estimate" package was utilized to analyze the immune score and stromal score of tumor samples. The objective was to examine the association between RIPK2 expression level and the estimated immune score in TCGA-pan cancers. Heat Lollipop diagrams were employed to visually represent the different cancer types on the vertical axis, immune scores on the horizontal axis, and correlation scores (*P<0.05, **P<0.01). Furthermore, the relationship between RIPK2 expression and infiltration of six types of immune cells, namely B cells, cluster of differentiation 4+ (CD4+) T cells, CD8+ T cells, neutrophils, macrophages, and dendritic cells (DCs), was investigated.

Furthermore, a correlation was observed between the expression of RNA modification genes, immune immunomodulatory genes, immune checkpoint-related genes, and the RIPK2 gene in STAD. The correlations were analyzed using Pearson's correlation coefficient. The heat maps depict the cancer types on the vertical axis, the immune scores on the horizontal axis, and the correlation scores. Statistical significance was indicated by *P<0.05 and **P<0.01.

### Relationship between RIPK2 gene expression and TMB or MSI or tumor stemness

Pearson's correlation analysis was employed to investigate the association between the expression of RIPK2 and tumor mutational burden (TMB), microsatellite instability (MSI), or tumor stemness. The expression data of RIPK2 obtained from TCGA was correlated with TMB, MSI, or tumor stemness (correlation coefficient) on the x-axis, while cancer types were represented on the y-axis.

### Cell culture and cell transfection

HGC-27 cells were cultured in DMEM/F12 medium (SH30023; HyClone, United States) containing 10% fetal bovine serum (FBS, sijiqing, Hangzhou, China) at 37◦C in a humidified atmosphere of 5% CO2. LV3 (H1/GFP&Puro)-RIPK2 lentivirus, which was constructed by Suzhou GenePharma Co., Ltd., were used to transfect HGC-27 cells. Lentiviruses were transfected with MOI 100, and 48 hours after transfection, cells were selected by puromycin to generate stable cell lines (shRNA-RIPK2), HGC-27 cell lines transfected with the lentiviral vector were used as negative control (NC) group.

### RT-qPCR

Total cell RNA was extracted using Trizol reagent (15596-026; Invitrogen) and then reversetranscribed complementary DNA (cDNA) was synthesized using a PrimeScriptTMRT reagent Kit with gDNA Eraser (RR047A; Takara), following the manufacturer's instructions as previous described 39. Primer used in this study: RIPK2, sense: 5- GAATCATGTGGATCCTCTCAGC-3; anti-sense: 5′-TGATTTCCAGGACAGTGATGC-3′. GAPDH, sense: 5′-CATCATCCCTGCCTCTACTGG-3′; anti-sense: 5′-GTGGGTGTCGCTGTTGAAGTC-3′. 2^-△△^CT method was used to calculate the relative RIPK2 mRNA expression.

### Tumorigenicity assays

The Animal Ethics and Use Committee of Renmin Hospital of Wuhan University approved the tumor-forming experiment in nude mice. To verify whether RIPK2 could affect the growth of cancer cells* in vivo*, xenograft tumors were established in nude mice. This study was validated with GC as a representative. Ten specific-pathogen-free male BALB/c nude mice (aged 4-6 weeks) were from Beijing Vital River Laboratory Animal Technology Co., Ltd., and the mice were randomly assigned to two groups (sh-RIPK2 and NC group) with 5 mice in each group. The tumor xenografts were developed by subcutaneously injecting approximately 1×10^6^ transfected HGC-27 cells suspended in 100 μL medium, into one flank of the nude mice to develop tumor (5 mice per group). The nude mice were killed on the 36th day, and the transplanted tumors were taken out, and the weights of xenograft tumors and nude mice were measured.

### Immunohistochemistry

For IHC, an UltraSensitiveTM SP (Mouse/Rabbit) IHC Kit (Maxim; Fuzhou, China) was used following the manufacturer's instructions. Transplanted tumor tissues of nude mice were embedded in paraffin. After deparaffinization, hydration, antigen retrieval, and serum block, tumor sections were incubated overnight with the following primary antibodies at 4°C: RIPK2 (1:1100, catalog no.DF6967, Affinity, Melbourne, Australia) and PCNA (1:400, catalog no. BM0104, Boster, Wuhan, China). Species-specific secondary antibodies were added to the slices. All samples were analyzed three times.

### TUNEL assay

TUNEL staining was performed using the one step TUNEL Apoptosis Kit (Beyotime, Hangzhou, China; catalog no: C1090). After the tumor sections were deparaffinized and rehydrated, the proteinase K (3315836001, Roche, Switzerland) was added for incubation. Tissues were counterstained with 4′, 6-diamidino-2-phenylindole (DAPI). Fluorescent images were acquired with a fluorescence microscope. All the specimens were processed into 3 sections, in each of the sections, 10 non-overlapping fields were randomly selected to count the number of cells with dyed and visible nuclei. The quantification of positive cells was obtained by Image-J. All samples were analyzed three times.

### Statistical Analysis

All statistical analyses were performed using software R (version 3.6.1). Differences in quantitative data between the two groups were analyzed using paired or unpaired Student's *t*-test, Mann-Whitney U-test, or Dunnett's t-test as appropriate. The immune checkpoint expression, immunomodulatory genes expression and levels of immune infiltration were determined using Pearson's correlation test. We considered a *P* < 0.05 as statistically significant (*P < 0.05, **P < 0.01, ***P < 0.001, ****P < 0.0001).

## Results

### The number of literatures of RIPKs family genes in cancer

**Figure 2:**
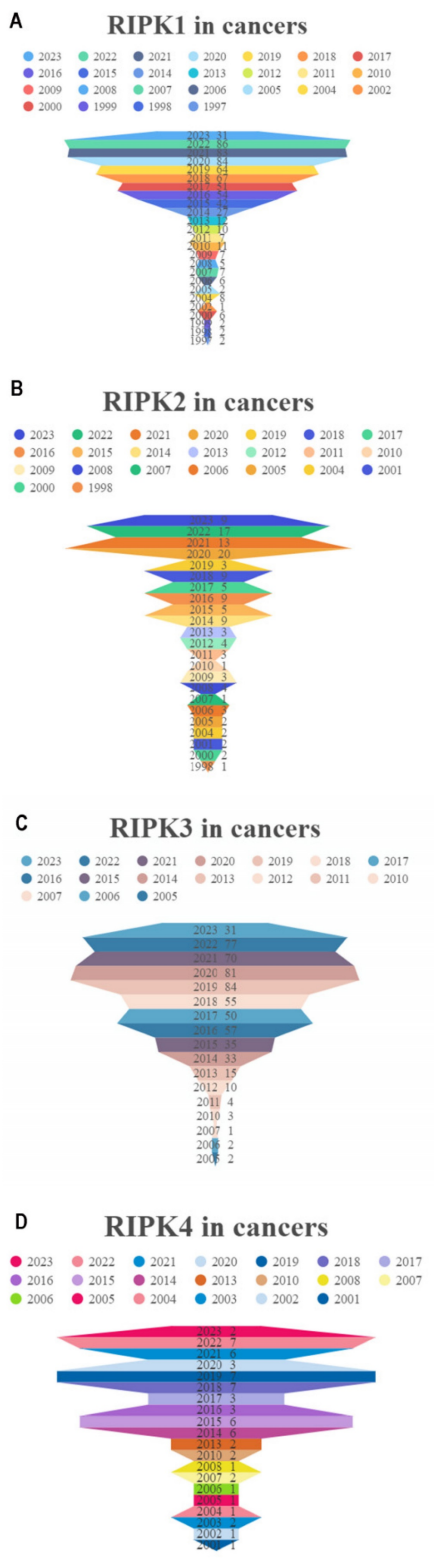
Researches on four RIPK family genes in relation to cancers. (A-D) The annual publication counts of RIPK1, RIPK2, RIPK3, and RIPK4 in relation to tumors was determined using the PubMed database.

The development of cancer has been the subject of extensive research, with a total of 615 papers examining the effects of RIPK1, 119 papers investigating the effects of RIPK2, 550 papers exploring the effects of RIPK3, and 54 papers analyzing the effects of RIPK4 (**Figure 2A-D**). These results indicate that RIPK family genes play an important role in the development of tumors.

### RIPK family genes mRNA expression levels in pan-Cancer

**Figure 3:**
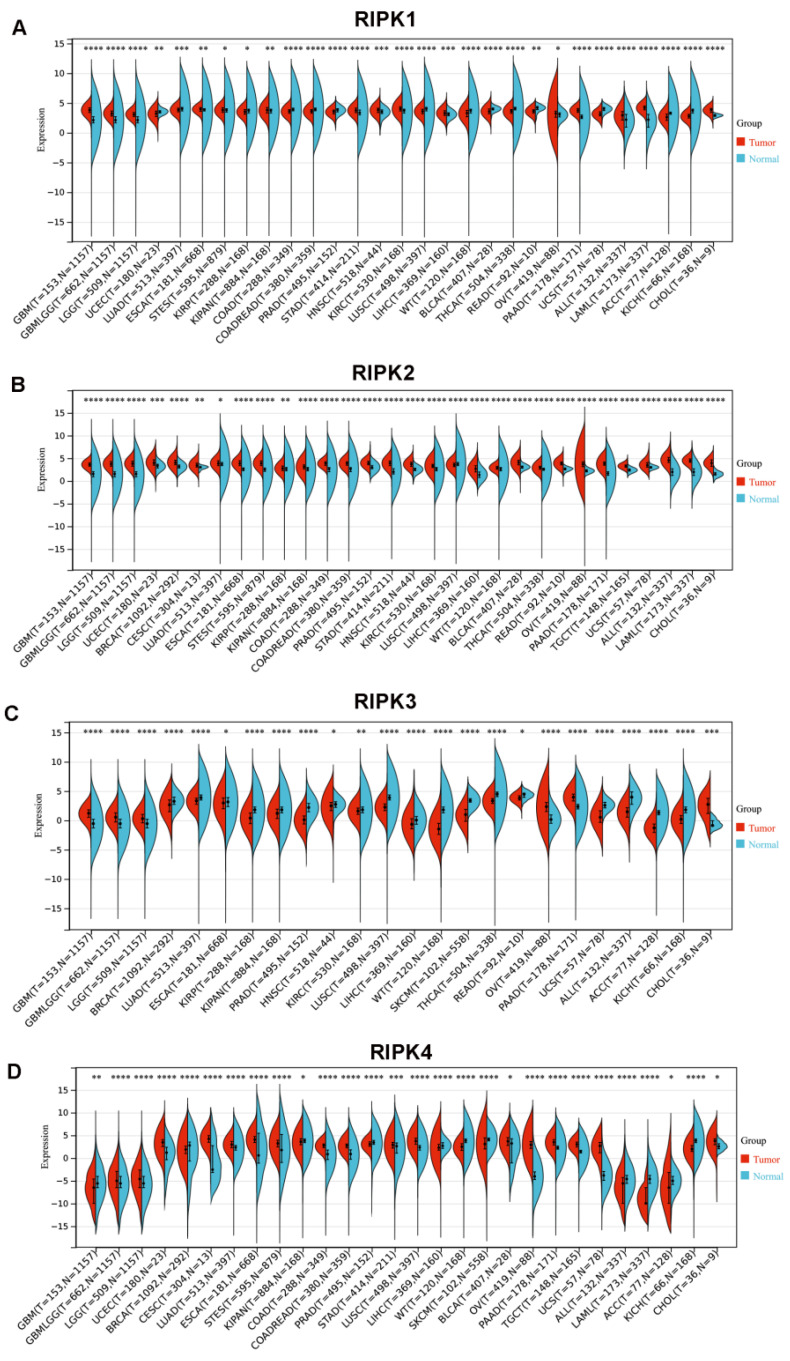
mRNA expression of four RIPK family genes in pan-cancer. (A-D) Differential expression of RIPK1, RIPK2, RIPK3 and RIPK4 in human tumors and normal tissues using data from TCGA. * P<0.05 was recognized as statistically significant. TCGA, The Cancer Genome Atlas.

In comparison to corresponding normal tissues, RIPK1 exhibits elevated expression levels in the majority of cancers, with the exception of colon adenocarcinoma (COAD), rectum adenocarcinoma (READ), prostate adenocarcinoma (PRAD), kidney renal clear cell carcinoma (KIRC), bladder urothelial carcinoma (BLCA), lung squamous cell carcinoma (LUSC), adrenocortical carcinoma (ACC), and kidney chromophobe (KICH) (**Figure 3A**). Conversely, RIPK2 demonstrates heightened expression levels in most cancers (**Figure 3B**). RIPK3 exhibits reduced expression levels in the majority of cancers, with the exception of Glioblastoma (GBM), Ovarian Cancer (OV), pancreatic adenocarcinoma (PAAD), and cholangiocarcinoma (CHOL), where it demonstrates elevated expression levels compared to their respective normal tissues (**Figure 3C**). Conversely, RIPK4 displays heightened expression levels in most cancers, except for GBM, breast cancer (BRCA), liver hepatocellular carcinoma (LIHC), skin cutaneous melanoma (SKCM), ACC, and KICH, where it exhibits decreased expression levels relative to normal tissues (**Figure 3D**). The cancer names and their corresponding abbreviations can be found in the Abbreviations section.

### Promoter methylation level of RIPKs in gastric cancer

**Figure 4:**
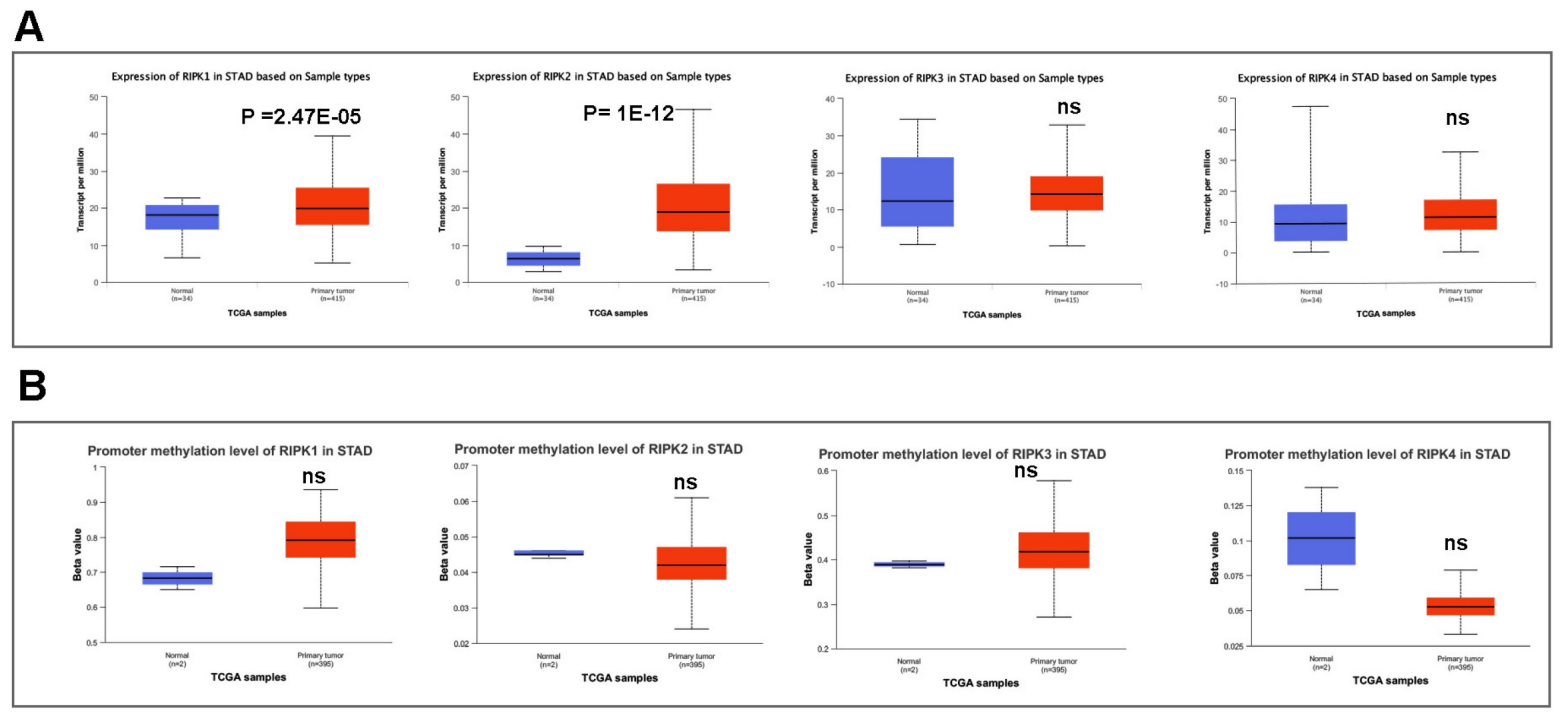
The mRNA expression and promoter methylation level of four RIPK family genes in GC. (A) RIPK1, RIPK2, RIPK3 and RIPK4 mRNA expression in GC tissues and normal tissues. (B) Promoter methylation level of RIPK1, RIPK2, RIPK3 and RIPK4 in GC. * P<0.05 was recognized as statistically significant. GC, gastric cancer.

We subsequently conducted a comparison of the disparities in promoter methylation levels of RIPKs between normal tissues and primary tumor tissues. Notably, **Figure 4A** exhibited a noteworthy upregulation in mRNA expression of RIPK1 (*P* = 2.47E-05) and RIPK2 (*P* =1E-12) in GC cases. Conversely, there was no discernible variation in mRNA expression of RIPK3 and RIPK4 in GC tissues. Additionally, we observed an elevation in promoter methylation levels of RIPK1 and RIPK3 in GC, while a reduction in promoter methylation levels of RIPK2 and RIPK4 was detected in GC (**Figure 4B**). However, the lack of adequate normal control samples prevented the establishment of statistical significance.

**Figure 5:**
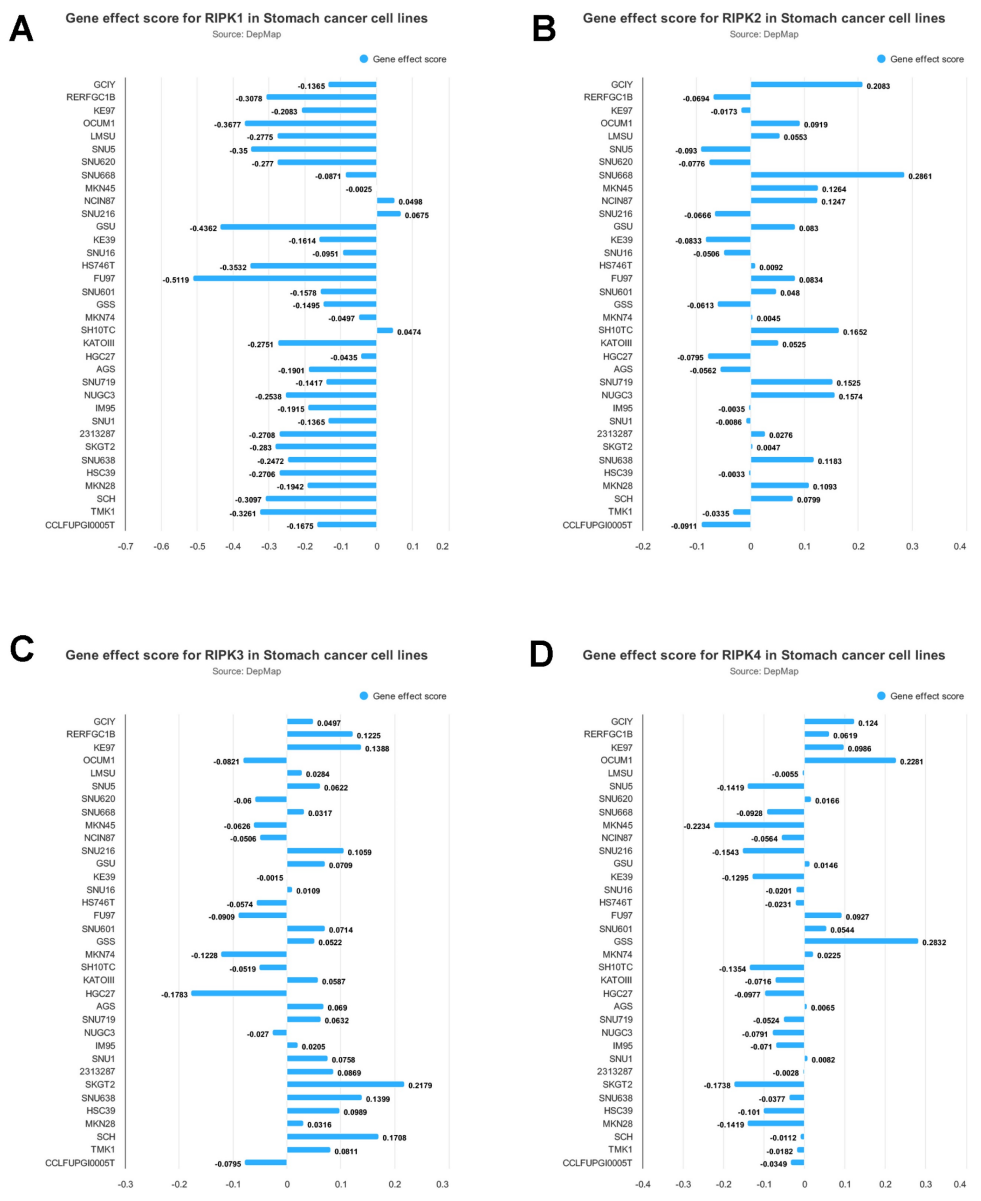
Gene effect score for RIPK1(A), RIPK2 (B), RIPK3(C) and RIPK4 (D) in GC cell lines. Negative scores indicate the inhibition of cell growth resulting from the knockout of specific genes.

The impact of RIPKs genes on the proliferation of gastric cancer cells is illustrated in **Figure 5**. The experimental findings demonstrate that the inhibition of RIPK1 significantly impedes the growth of FU97 cells. Conversely, the deletion of RIPK2 notably enhances the growth of SNU668 cells, while the deletion of RIPK3 promotes the growth of SKGT2 cells. Similarly, the deletion of RIPK4 leads to a significant promotion in the growth of GSS cells. Consequently, this analysis enables the preliminary identification of cells suitable for studying the associated genes.

### Correlation between RIPK2 expression and immune infiltration

**Figure 6:**
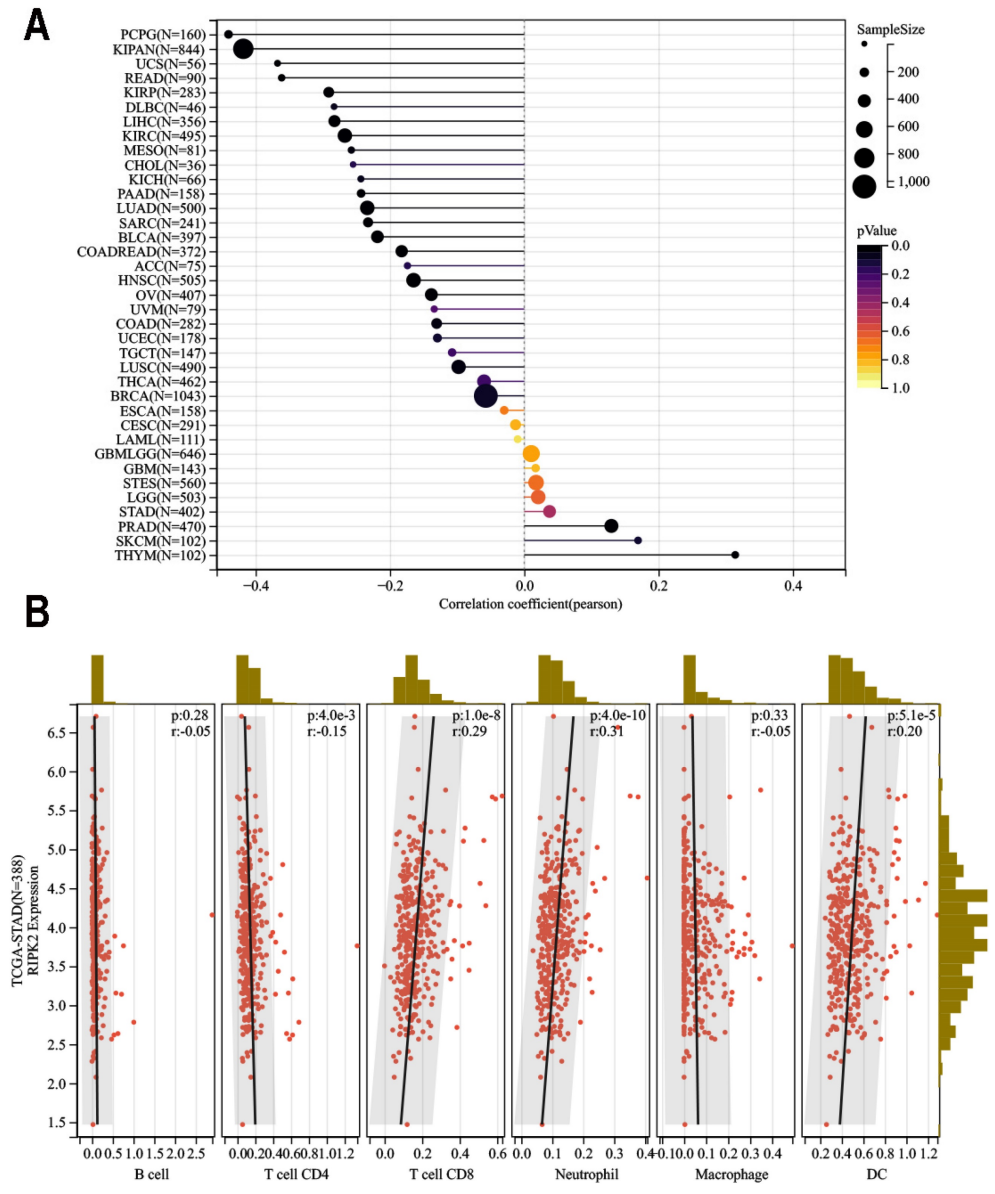
Relationship between RIPK2 expression and immune infiltration in pan-cancer. (A)RIPK2 expression was significant correlations with majority tumors immune infiltration; (B)RIPK2 expression was significantly correlated with CD4^+^Tcell, CD8^+^Tcell, neutrophil and DC infiltration in GC. * P<0.05 was recognized as statistically significant.

The impact of immune and stromal cells within the tumor microenvironment on patient survival has been demonstrated, thereby providing evidence for the prognostic significance of RIPK2 across various cancer types. Consequently, it would be worthwhile to explore the association between RIPK2 expression and immune infiltration. In this study, we conducted a comprehensive analysis of RIPK2 expression within the tumor microenvironment, utilizing the immune score and stromal score as indicators (**Figure 6A**).

Our findings revealed a significant positive correlation between RIPK2 expression and infiltration of CD8+ T cells (R = 0.29, *P* = 1.0E-8), Neutrophil (R = 0.31, *P* = 4.0E-10), and dendritic cells infiltration (R = 0.20, *P* = 5.1E-5). In contrast, a negative correlation was observed between RIPK2 expression and CD4 infiltration (R = 0.15 *P* = 4.0E-3). Conversely, no statistically significant correlation was found between Macrophage (R = 0.33 *P* = 4.0E-3) and B cell infiltration in GC (**Figure 6B**).

**Figure 7:**
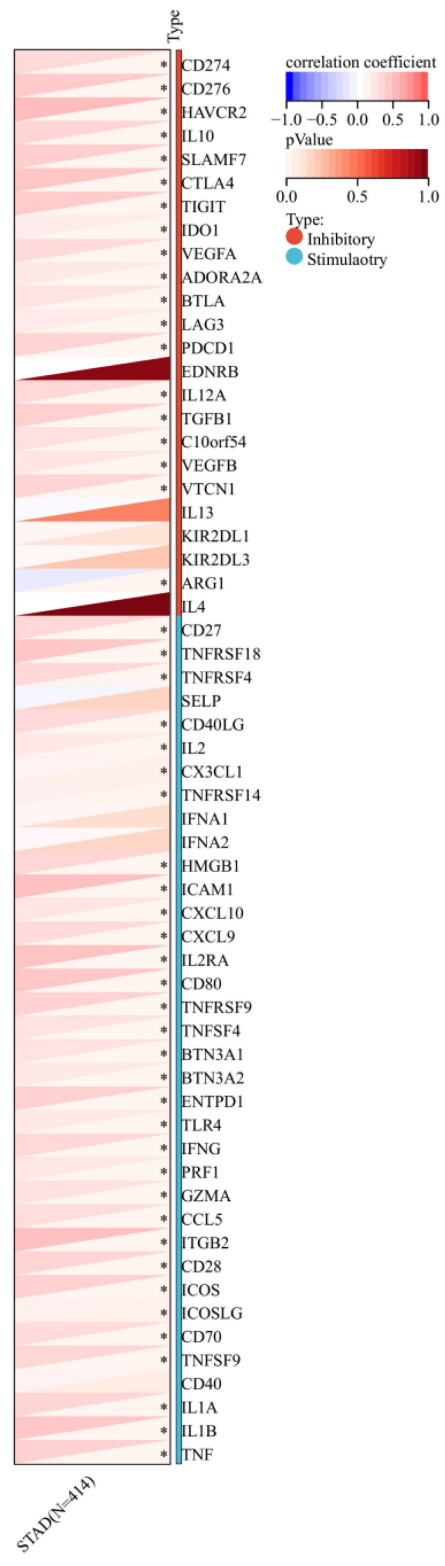
The association between the expression of RIPK2 and immune checkpoint genes. * P<0.05 was recognized as statistically significant.

**Figure 8:**
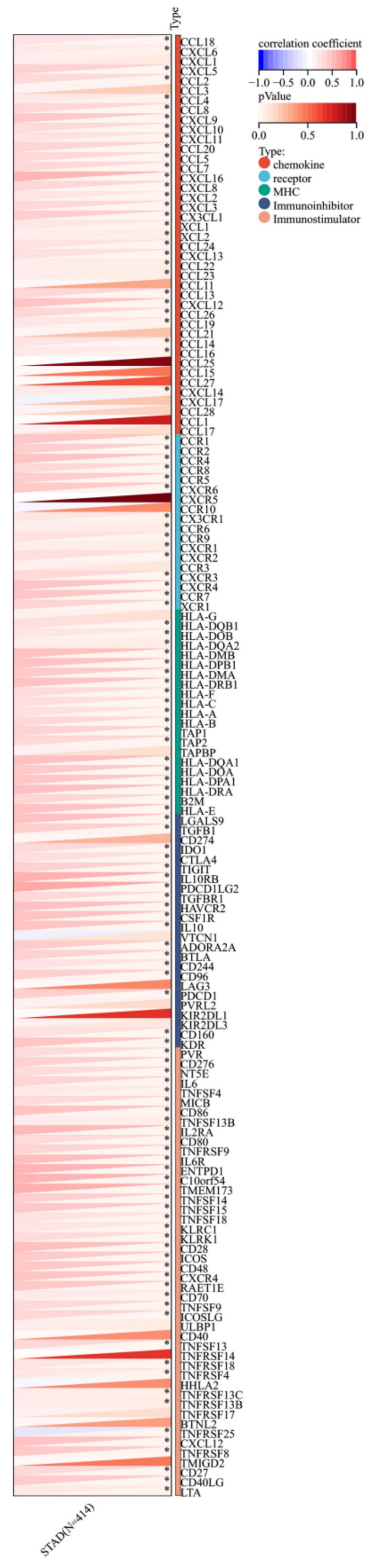
Correlation of RIPK2 expression with immune modulatory genes, chemokine receptors, major histocompatibility complex (MHC) genes, immunoinhibitory genes and immunostimulatory genes. * P<0.05 was recognized as statistically significant.

Furthermore, a significant association was observed between the expression of RIPK2 and immune checkpoint genes, including CD274, CD276, and IL-10 (**Figure 7**). Additionally, a strong correlation was found between the expression of RIPK2 and immune modulatory genes, such as chemokines (CCL8, CXCL6, et al.), chemokine receptors (CCR1, CCR2, et al.), major histocompatibility complex (MHC) genes (HLA-DQB1, HLA-DOB, et al.), immunoinhibitors (TGFB1, CTLA4, et al.), and immunostimulators (CD276, IL6, et al.) (**Figure 8**).

### Association of RIPK2 with RNA methyltransferase and heterogeneity analysis

**Figure 9:**
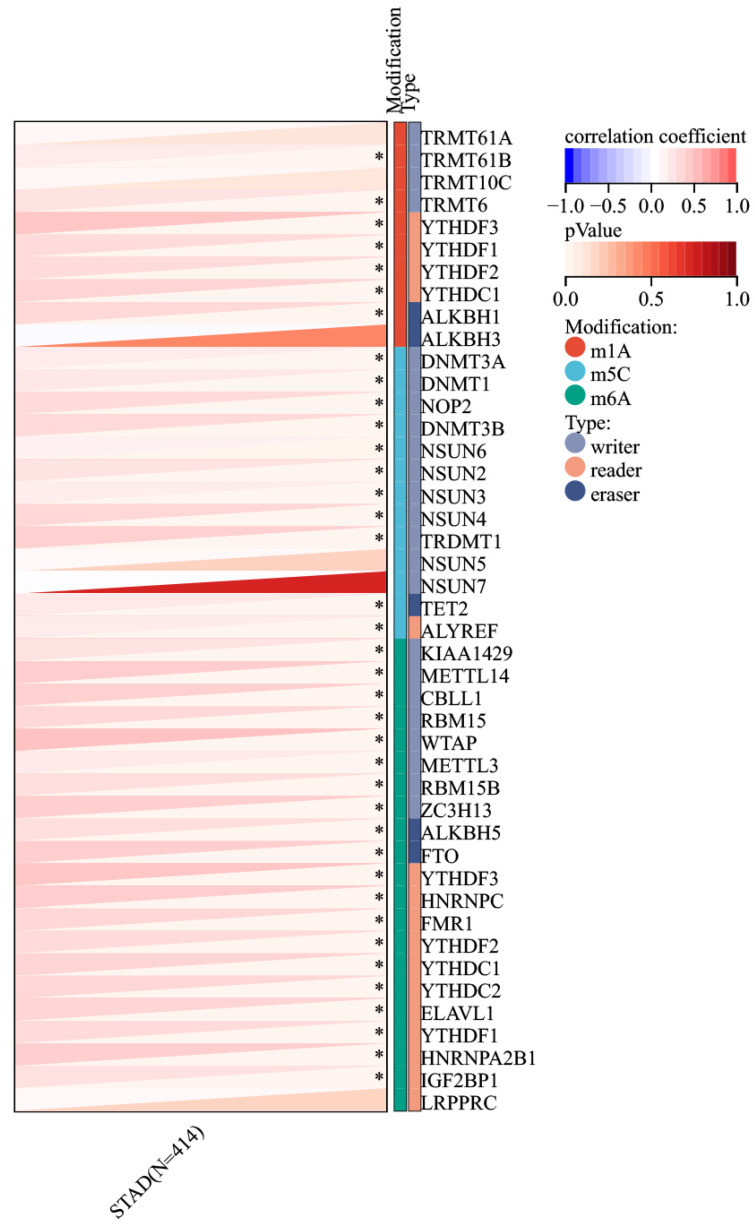
Correlation of RIPK2 expression with RNA methyltransferase. * P<0.05 was recognized as statistically significant.

Given the increased expression of RIPK2 RNA in GC compared to adjacent tissues, an investigation was conducted to explore the correlation between RIPK2 and RNA methylation enzymes. The findings revealed a positive association between RIPK2 RNA expression and a majority of methylation enzymes in GC (**Figure 9**). Additionally, the study examined the relationship between RIPK2 expression and two novel immunotherapy response biomarkers, namely MSI and TMB. The results indicated that RIPK2 expression was significantly linked to MSI in 33 different cancer types, with negative correlations observed in DLBC and KIPAN, and positive correlations observed in BRCA, BLCA, STAD, UCEC, THYM, STES, and SARC (**Supplementary Figure 1A**). The positive association between RIPK2 expression and TMB has been observed in various tumors, such as CHOL, KIRP, BRCA, KIPAN, BLCA, LUSC, LUAD, SARC, STAD, UCS and THYM (**Supplementary Figure 1B**). The presence of tumor cells exhibiting stemness characteristics poses a challenge to effective tumor eradication, leading to the exclusion of compounds that are inversely correlated with stemness from tumor therapy. Consequently, the relationship between RIPK2 expression and tumor stemness was investigated. The findings indicated a negative association between RIPK2 expression and tumor stemness in PCPG, LGG, LUSC, LIHC, KIRC and HNSC, while a positive correlation was observed in other contexts (**supplementary Figure 1C**). These results suggest that RIPK2 may have implications for subsequent treatment strategies in tumor progression.

### Knockdown of RIPK2 inhibits gastric cancer growth in vivo

**Figure 10:**
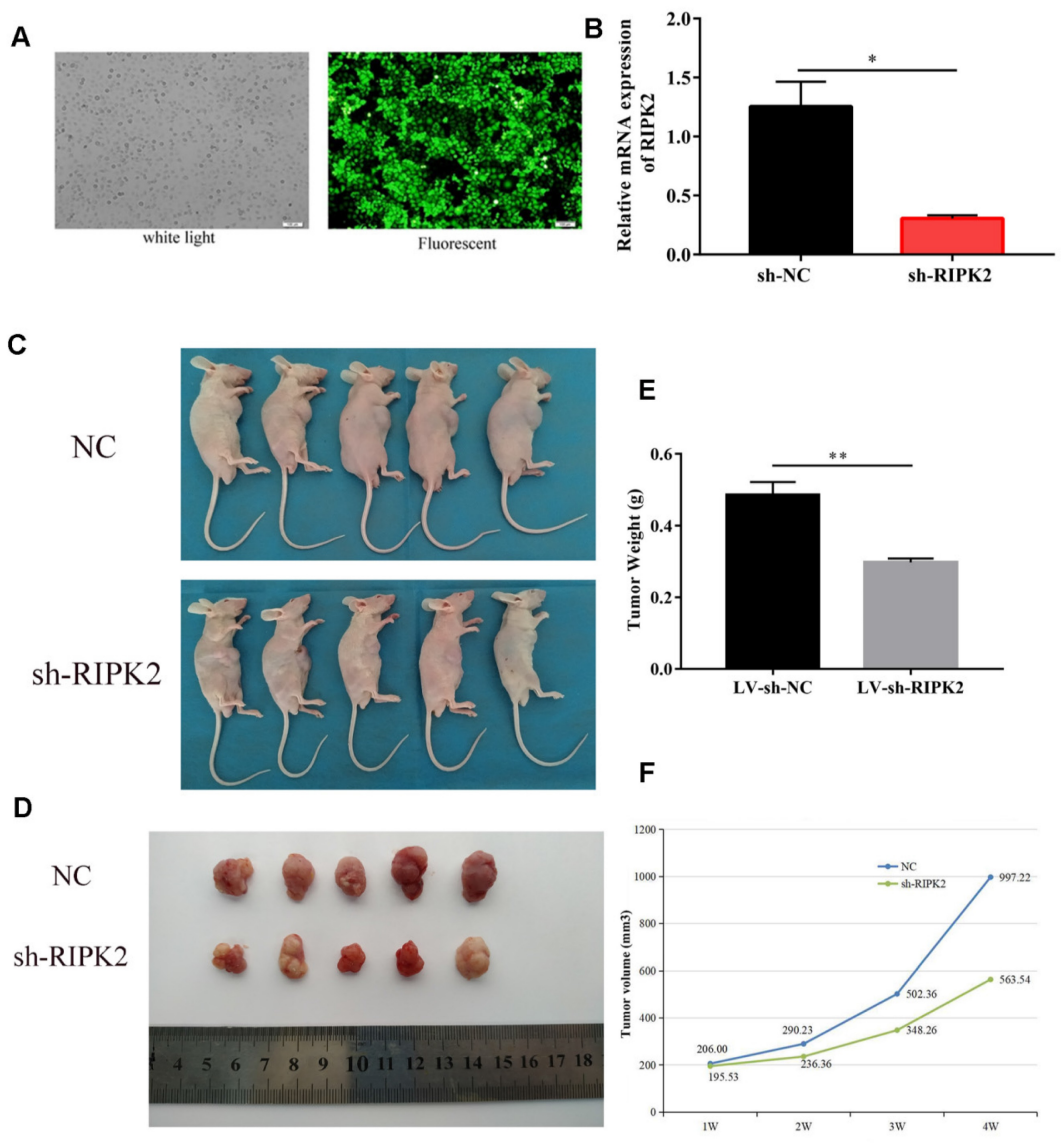
knockdown of RIPK2 inhibits GC growth *in vivo.* (A-B) the HGC-27 cells transfected with shRNA-RIPK2, transfection efficiency was detected by fluorescent and qRT-PCR assays. The cells transfected with empty plasmid were used as negative control. (C-D) Mice were euthanized and tumors obtained from mice on day 36 after injection. (E-F) Tumors weight and volume were measured in two groups.

To investigate the effects of RIPK2 on tumor cells growth, we choose GC cells to demonstrate it. HGC-27 cells were transfected with shRNA-RIPK2, and then the silencing transfection efficiency was detected by fluorescent and qRT-PCR assays (**Figure 10A-B**). Then, HGC-27 cells transfected with shRNA-RIPK2 or empty plasmid (negative control) respectively injected into one flank of the nude mice to develop xenograft tumor. 36 days after injection, mice were euthanized and tumors were taken out. It was shown that compared to NC group, silencing of RIPK2 could significantly suppress GC cells growth *in vivo* (**Figure 10 C-D**). As expected, the tumor weight and volume of sh-RIPK2 group were lighter and smaller than NC group (**Figure 10 E-F**).

**Figure 11:**
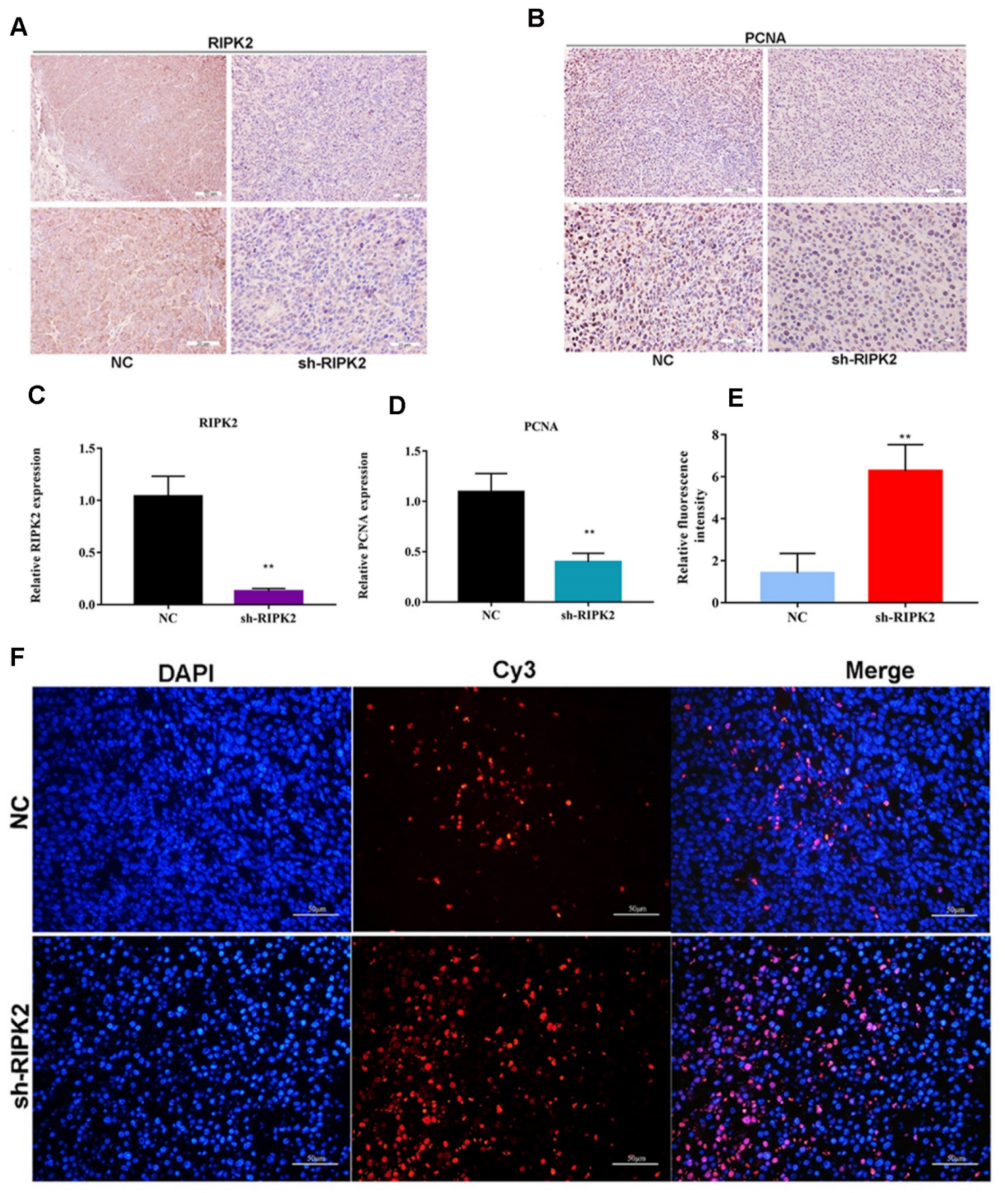
knockdown of RIPK2 suppressed GC cell proliferation and apoptosis* in vivo.* (A-D) IHC was used to detect the expression of RIPK2 and PCNA in tumor section, and quantification. (E-F) TUNEL was performed to detect the apoptosis cells in tumor section, and the quantification of fluorescent intensity. *P < 0.05, **P < 0.01.

Additionally, the proliferating cell nuclear antigen (PCNA) antibody was applied to detect the PCNA expression within two groups using IHC assay. It was indicated that the expression of PCNA was lower in RIPK2-silenced group tumor section (**Figure 11 A-D**), which suggested that knockdown of RIPK2 might inhibit GC cells growth by affecting PCNA. Furthermore, TUNEL assay was performed to detect the apoptosis cells in both two groups' tumor section, and it was shown that the quantification of red fluorescent intensity in RIPK2-silenced group is higher (**Figure 11 E-F**), which demonstrated that knockdown of RIPK2 promotes the GC cell apoptosis* in vivo*.

## Discussion

The involvement of RIPK family genes in cancer progression is of significant importance. A comprehensive search on PubMed revealed a growing number of articles investigating RIPK family genes, particularly RIPK1, RIPK2, RIPK3 and RIPK4, in the context of tumor research. Moreover, our analysis of TCGA-pan cancer data demonstrated differential expression of RIPK family genes in various tumor tissues, including GC. Consequently, this study aims to specifically examine the impact of RIPK family genes in GC. Promoter methylation is correlated with both the loss of expression and the advanced stage of the disease. Additionally, promoter methylation serves as an additional mechanism for the suppression of tumor suppressor genes in cancer. However, due to the limited sample size, our study was unable to yield significant data. In future research, we anticipate expanding the sample size to facilitate a more comprehensive analysis of the expression of promoter methylation in the RIPK family genes in GC.

Previous studies have demonstrated the involvement of RIPK2 in various cellular biological processes across diverse species, including gene transcription regulation, inflammatory and immune responses, autophagy, and apoptosis 40-42. Recent investigations have additionally identified functional connections between RIPK2 and clinical diseases, notably tumors 6, 22, 43. Nevertheless, the extent to which RIPK2 contributes to the pathogenesis of distinct tumors via shared molecular mechanisms remains unexplored. Consequently, we have chosen to conduct further analysis on RIPK2.

In recent times, there has been a growing scholarly interest in the tumor microenvironment 44, with a particular focus on the crucial roles played by immunity and metabolism. Earlier investigations have demonstrated that the promotion of bone marrow mesenchymal stem cells' growth is influenced by RIPK2, which in turn affects lymphocyte infiltration and metastasis of BLCA 24. Furthermore, RIPK2's regulatory influence on the gastric mucosal immune system suggests a potential association with an increased risk of GC 22. Macrophages, CD4^+^ T cells, B cells, CD8^+^ T cells and neutrophils are found in the TME, and involve in the occurrence of cancer 33. Our findings demonstrate a significant association between RIPK2 and the degree of immune cell infiltration within certain tumor microenvironments.

The potential correlation between immune checkpoint genes and genes that facilitate immune cell infiltration is worth exploring. Complex interactions between the immune system and cancer, including the manipulation of immune checkpoints such as programmed cell death 1 (PD-1), enable tumor cells to evade immune surveillance 45. Notably, cytotoxic T-lymphocyte-associated protein 4 (CTLA4) and PD-1 (PDCD1) genes serve as exemplars of immune checkpoint genes, and the application of antibody therapy to inhibit the CTLA4 or PD-1 checkpoints has been shown to unleash T-cells for tumor eradication 46. Within our study, we have observed a positive association between RIPK2 expression and the expression of CTLA4 and PDCD1, which may offer valuable insights into the development of immune checkpoint inhibitors. In the present study, we also observed a correlation between RIPK2 and immune checkpoint inhibitors immunotherapy-related biomarkers, including TMB, MSI, and tumor stemness. Previous research has demonstrated that TMB is indicative of immunotherapy efficacy, with higher TMB values associated with greater tumor inhibition and clinical benefits resulting from immunotherapy. Conversely, lower TMB values are associated with diminished clinical benefits derived from immunotherapy 47. The study demonstrated a positive correlation between the expression of RIPK2 and the TMB and MSI in different types of tumors, including stomach adenocarcinoma (STAD). Consequently, elevated levels of RIPK2 expression could potentially serve as a predictive factor for significant responses to checkpoint inhibitors in GC.

The role of cancer stemness in tumor initiation, progression, and drug resistance has been well-established, with regulation potentially occurring through the TME and intrinsic plasticity of tumor cells. Previous studies have shown that RIPK2 promotes the development of metastatic tumor tissues 6 and inflammatory breast cancer (IBC) 43, while the suppression of RIPK2 can hinder the migration of BC cells and reduce the occurrence of extrapulmonary metastasis 48. Furthermore, available evidence suggests that RIPK2 plays a crucial role in chemoresistance in IBC and that inhibiting RIPK2 may be a promising approach to decrease the recurrence of IBC 43. Therefore, it is hypothesized that the regulation of tumor stemness by RIPK2 may have an impact on cancer progression, necessitating further investigation.

Additionally, the emergence of m6A, m1A, and m5C as crucial modulators in various biological processes highlights the potential of targeting RNA methylation at these sites for therapeutic purposes 49-51. In this study, a significant correlation between RIPK2 expression and RNA methylation enzymes in GC was observed, warranting additional research to validate these findings. Consequently, these results suggest that RIPK2 shows promise as a potential target for tumor immunotherapy in GC; however, further confirmation is necessary.

RIPK2 has been implicated in the regulation of chronic viral hepatitis B development 52 and the facilitation of migration and metastasis in LIHC cells 25. Our previous investigation has confirmed that the inhibition of RIPK2 suppresses the growth of GC cells by modulating IKBα/NF-κB signaling 53. Consequently, RIPK2 emerges as a promising target for cancer treatment 54 and autoimmune diseases 55. Based on the findings of our* in vivo* experiment, we have successfully illustrated that the suppression of RIPK2 effectively impedes the proliferation of tumor cells *in vivo* through its influence on the cell cycle-associated protein PCNA and apoptosis. This discovery provides a significant avenue for our future investigations into the role of RIPK2 in the intricate process of GC development, and the subsequent phase entails conducting a more comprehensive investigation of RIPK2 within the context of immunotherapy.

However, it is important to acknowledge the limitations of our study. Firstly, the absence of adequate research funds prevented us from conducting *in vivo* research experiments on the four genes belonging to the RIPKs family. Secondly, our investigation solely relied on lentivirus-mediated knockdown of the RIPK2 for tumorigenesis experiments. In future endeavors, we intend to undertake comprehensive research utilizing RIPK2 inhibitors to ascertain the potential transformation of RIPK2 into a viable treatment for tumors.

## Conclusion

In summary, our study indicated that RIPK2 is an immune-related gene. Suppression of RIPK2 effectively inhibits the growth of GC cells. RIPK2 exhibits potential as a feasible target for immunotherapy in diverse cancers.

## Supplementary Material

Supplementary figure.

## Abbreviations

ACC: Adrenocortical carcinoma
BC: Breast cancer
BLCA: Bladder Urothelial Carcinoma
BRCA: Breast invasive carcinoma
CESC: Cervical squamous cell carcinoma and endocervical adenocarcinoma
CHOL: Cholangiocarcinoma
COAD: Colon adenocarcinoma
READ: Rectum adenocarcinoma
DLBC: Diffuse Large B-cell Lymphoma
ESCA: Esophageal carcinoma
GBM: Glioblastoma multiforme
HNSC: Head and Neck squamous cell carcinoma
KICH: Kidney Chromophobe
KIRC/CRCC: Kidney renal clear cell carcinoma/ Renal clear cell carcinoma
KIRP: Kidney renal papillary cell carcinoma
LAML: Acute Myeloid Leukemia
LGG: Brain Lower Grade Glioma
LIHC: Liver hepatocellular carcinoma
LUAD: Lung adenocarcinoma
LUSC: Lung squamous cell carcinoma
MESO: Mesothelioma
OV: Ovarian serous cystadenocarcinoma
PAAD: Pancreatic adenocarcinoma
PCPG: Pheochromocytoma and Paraganglioma
PRAD: Prostate adenocarcinoma
READ: Rectum adenocarcinoma
SARC: Sarcoma
SKCM: Skin Cutaneous Melanoma
STAD: Stomach adenocarcinoma
TGCT: Testicular Germ Cell Tumors
THCA: Thyroid carcinoma
THYM: Thymoma
UCEC: Uterine Corpus Endometrial Carcinoma
UCS: Uterine Carcinosarcoma
UVM: Uveal Melanoma
