# Supplementary material for: Receptor-interacting protein kinase 2 is associated with tumor immune infiltration, immunotherapy-related biomarkers, and affects gastric cancer cells growth in vivo
Source: J Cancer. 2024 Jan 1;15(1):176–91. doi: 10.7150/jca.90008 (PMC10751663; doi:10.7150/jca.90008)
Supplement: Supplementary file 1 — Supplementary figure. [file jcav15p0176s1.pdf]

Supplementary Figure 1

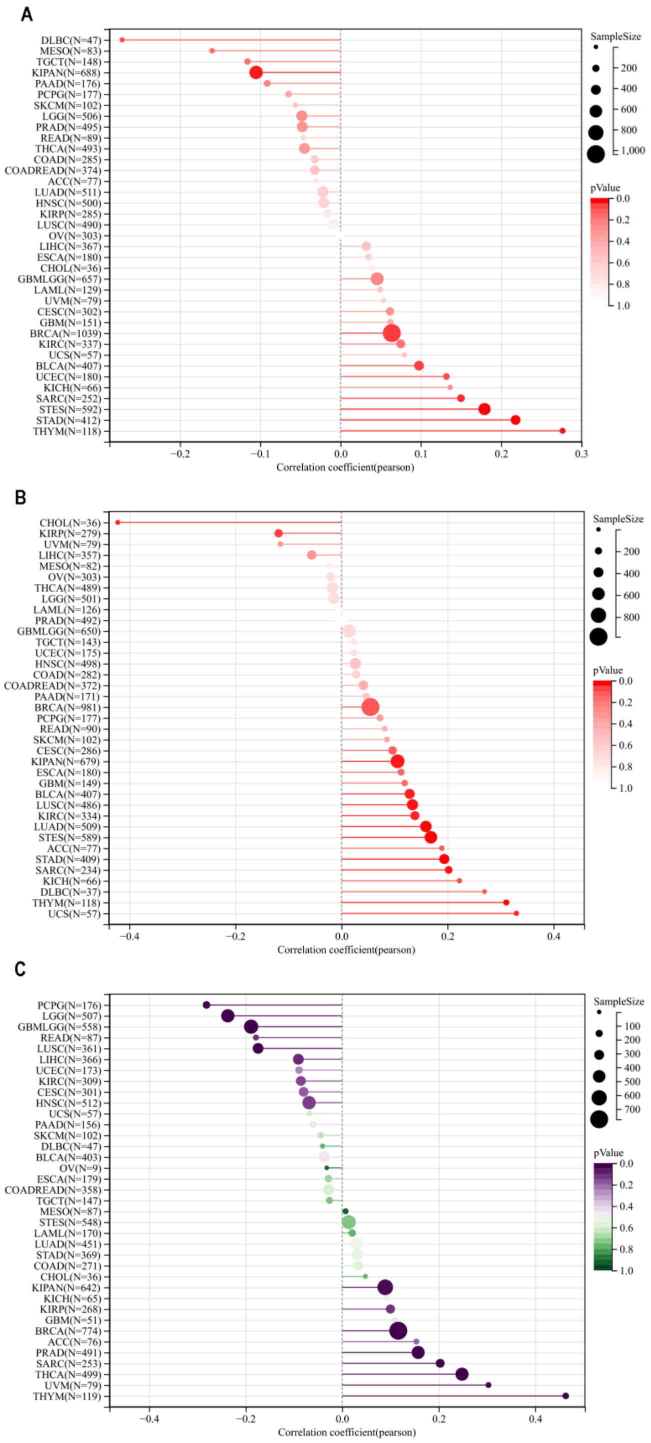

Supplementary Figure 1 Relationship between RIPK2 expression and MSI (A), TMB (B) and tumor stemness (C). Cancer types on the vertical axis, the immune scores on the horizontal axis, and the correlation scores.
